# Correlations between opioid mortality increases related to illicit/synthetic opioids and reductions of medical opioid dispensing - exploratory analyses from Canada

**DOI:** 10.1186/s12889-020-8205-z

**Published:** 2020-01-31

**Authors:** Benedikt Fischer, Wayne Jones, Mark Tyndall, Paul Kurdyak

**Affiliations:** 1 Schools of Population Health and Pharmacy, Faculty of Medical and Health Sciences, University of Auckland, 85 Park Rd, Grafton, Auckland, 1023 New Zealand; 2 Department of Psychiatry, University of Toronto, Toronto, ON Canada; 3 Centre for Applied Research in Mental Health and Addiction (CARMHA), Faculty of Health Sciences, Simon Fraser University, Vancouver, BC Canada; 4 Department of Psychiatry, Federal University of São Paulo (UNIFESP), São Paulo, Brazil; 5 School of Population and Public Health, University of British Columbia, Vancouver, Canada; 6 Institute for Mental Health Policy Research, Centre for Addiction and Mental Health, Toronto, ON Canada; 7 Institute for Clinical Evaluative Sciences (ICES), Toronto, ON Canada

**Keywords:** Prescription opioids, Mortality, Synthetic opioids, Non-medical use, Supply, Substitution, Public health, Canada

## Abstract

**Background:**

North America has been experiencing a persistent epidemic of opioid-related overdose mortality, which has increasingly been driven by fatalities from illicit, toxic opioids in most recent years. Patterns of synthetic opioid availability and related mortality are heterogeneous across Canada, and differing explanations exist as to their differentiated proliferation. We examined the perspective that heterogeneous province-based variations in prescription opioid availability, facilitated by various control strategies, post-2010 may have created regionally differential supply gaps for non-medical opioid use substituted by synthetic opioid products with differential impacts on mortality risks and outcomes in Canada.

**Methods:**

We examined annual, prescription opioid dispensing rates and changes in the ten Canadian provinces (for the periods of 1) 2011–2018, 2) ‘peak-year’-to-2018) in Defined Daily Doses/1000 population/day, derived from data from a large representative, stratified sample of community pharmacies projected to a Canada total. Annual, provincial opioid-related mortality rates and changes for years 2016–2018 were calculated from federal data. We computed correlation values (Pearson’s R) between respective province-based change rates for prescription opioid dispensing and opioid-related mortality for the two over-time scenarios.

**Results:**

All but one province featured reductions in prescription opioid dispensing 2011–2018; seven of the ten provinces had increases in opioid mortality 2016–2018. The correlation between changes in opioid dispensing (2011–2018) and in opioid-mortality (2016–2018) was *r* = 0.63 (df = 8, *p*-value: 0.05); the correlation was *r* = 0.57 (df = 8, p-value: 0.09) for changes in opioid dispensing ‘peak year’-to-2018, respectively.

**Conclusions:**

Quasi-significant results indicate that recent increases in opioid-related deaths driven by illicit, synthetic opioids tended to be larger in provinces where reductions in prescription opioid availability have been more extensive. It is a plausible explanation that these reductions created supply gaps for non-medical opioid use increasingly filled by illicit, synthetic opioids differentially contributing to opioid-related deaths, generating un-intended adverse effects for previous interventions. General prevention measures to reduce opioid availability, and targeted prevention for at-risk opioid users exposed to toxic drug supply may be include counteractive effects and require coordinated reconciliation.

## Background

North America continues to experience an unprecedented public health crisis involving an extensive toll of opioid-related poisonings and mortality. Concretely, there were 47,600 opioid-related deaths in the US in 2017, and 4460 opioid-related deaths in Canada in 2018. While patterns regionally differ in both countries, these mortality numbers represent ~ 10% increases over previous years as well as yet similar country-based population rates [1, 2].

Opioid-related mortality in North America – and Canada specifically – was originally driven by high, and rising levels of medical opioid dispensing; however, these ecological parameters have shifted in recent years. Specifically, following a variety of system-level interventions to restrain opioid use and harms (e.g., select opioid formulation control; restrictive prescription guidelines and monitoring; enforcement) and increasing public awareness post-2010, a deceleration in medical opioid dispensing - albeit with substantial regional variations – occurred across North America [3, 4]. Based on standardized population-level measures (e.g., morphine equivalents or defined daily doses/capita), medical opioid dispensing declined by about 20% during the period 2010/2011 to 2015/2016 in both the US and Canada [5, 6]. In Canada, these declines had further accelerated by 2018, with reductions of up to 50% in medical PO dispensing – a halving of the population-level flow of prescribed opioids within just a few years – in some Canadian provinces (e.g., British Columbia, where strict opioid prescribing standards based on US prescribing guidelines were introduced in 2016) [7, 8]. In addition, heroin vanished from most local drug markets amidst rising prescription opioid availability without substantial return.

The discrepant developments of decreasing opioid availability and increasing opioid mortality have been explained mainly with the recent proliferation and use of new illicitly produced, highly potent and toxic synthetic opioid products (e.g., fentanyl or fentanyl analogues) [9, 10] which have greatly amplified the risk for overdose and fatalities among non-medical users. Recently, synthetic opioids have been implicated in substantial but regionally inconsistent proportions of opioid-related deaths (province-based range: 5–88% in 2018) [1]. As local availability and contribution of illicit/synthetic opioids to mortality has varied across North America, key questions regarding the drivers of these inconsistent patterns exist. While some explain the arrival of illicit/synthetic opioids as an independent supply ‘wave’, other perspectives have suggested that they have proliferated primarily as a direct consequence of substantially reduced availability of medically-dispensed opioids for non-medical use (‘supply gap’ theory) [11–13]. This perspective has been corroborated by various data on non-medical opioid use trajectories initiated with prescription opioids and transitioning to riskier opioid use modes (e.g., injecting) and/or illicit/synthetic opioid products among sub-populations of users [14, 15].

In the context of the above dynamics, the ‘supply gap’ perspective may suggest that jurisdictions with larger reductions in prescription opioid dispensing (involving lesser availability of medical opioids and greater illicit opioid exposure) would experience larger increases in opioid-associated risk and mortality among non-medical users. To examine the above perspective, we explored associations between recent, provincial patterns of over-time changes in medical opioid dispensing and corresponding changes in opioid-related mortality across Canada in the post-2010 period.

## Methods

**Fig. 1:**
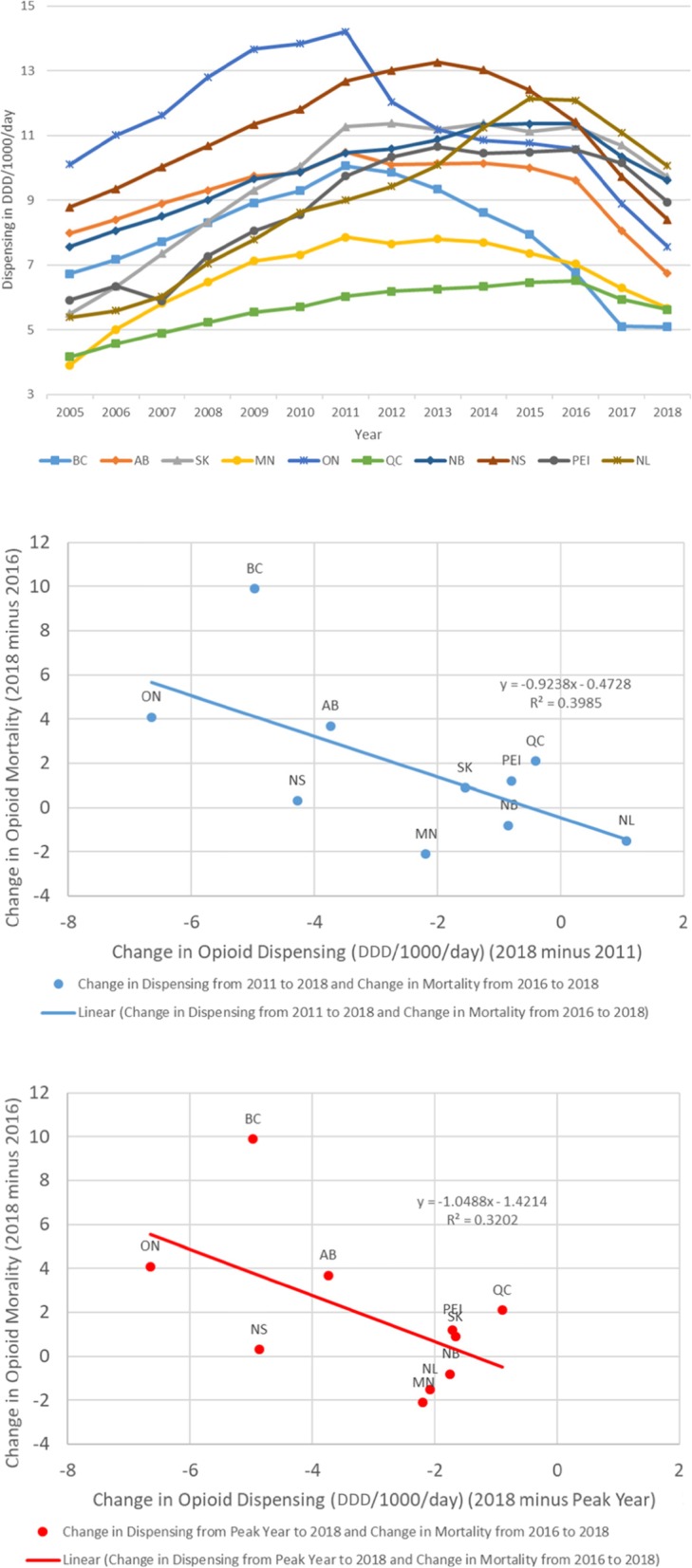
(1) Line-graph of province-based opioid dispensing (in DDD/1000/day) for years 2005–2018 and (2) scatterplots of correlations of changes in annual opioid dispensing (2a) 2018–2011 and (2b) 2018 – ‘peak year’ and opioid-related mortality (2018–2016) in Canada

Data used for analyses were obtained from two sources. First, medical opioid dispensing data came from previously examined information on community-based (retail) dispensing of prescribed opioid medications collected through a commercially assembled, stratified, representative (‘IQVIA’, formerly IMS Compuscript) pan-Canadian panel of about 6000 community-based pharmacies from which the total of national opioid dispensing in Canada is estimated by-way-of geospatial projection methodology, as used by similar drug utilization analyses [5, 16–18]. Original dispensing information included opioid product name, formulation, strength and dose, by province for the study period. Based on the WHO’s ‘Pain Ladder’ and Anatomical Therapeutic Chemical (ATC) classification, ‘strong opioids’ (i.e., excluding ‘weak’ opioids, as well as methadone due to inconsistent dispensing) were categorized and converted into annual Defined Daily Doses/1000 population/day (DDD/1000/day, a standard comparative drug utilization measure) values for the ten provinces for years 2011–2018 [19, 20]. Based on these annual, province-based opioid dispensing rates, we derived, a priori, two measures for subsequent analyses. The first measure was the differences in annual, province-based opioid dispensing (in DDD/1000/day) between 2011 and 2018; the second was the difference between each province’s ‘peak-year’ of opioid dispensing rate over the period 2011 to 2017 and the corresponding province’s 2018 rate. The rationale for these two measures was that 1) in Canada, key control interventions (e.g., select strong opioid delisting from public formularies, intensified prescription monitoring and /or guidelines for prescription opioids) targeting prescription opioid availability and use began in 2012 (i.e., with 2011 as last pre-intervention year), 2) opioid dispensing changes have varied substantially, e.g. in terms of timing, by province across Canada [5, 21] [see linegraph, Fig. 1].

Second, for opioid-related mortality we used available, annual population rates (per 100,000) of apparent opioid-related deaths, for the ten provinces, as reported by federal authorities for the three years 2016, 2017 and 2018 (only full years available for these data) [1]. The national opioid-related mortality data are based on investigations by provincial coroner services examining suspected unnatural deaths for drug-related causes. For this measure, the 2016 opioid-related mortality rate was subtracted from the 2018 rate for each province.

Based on the investigation’s focus on possible associations between the two above-defined outcomes, the statistic of interest was the Pearson product moment correlation values between the two sets of province-based changes in opioid dispensing rates [1] 2018–2011, and [2] 2018 -- provincial ‘peak year’ and the corresponding changes in annual, province-based opioid-related mortality rate (2018 to 2016). Correlation statistics were reported, and scatterplots for visualizations of the two correlations were generated. All analyses were computed in Microsoft Excel 2016.

No ethics review was required for this study due to the fully anonymous, de-personalized nature of the data used and analyses conducted.

## Results

[see Table 1, also for definition of acronyms of provinces, as well as linegraph, Fig. 1] For provincial, annual opioid dispensing, all but one province featured a decrease in rates over the period 2011–2018; all ten provinces featured a decrease when considering ‘peak year-to-2018’. ON had the highest opioid dispensing rate (14.2 DDD/1000/day), QC the lowest (6.0 DDD/1000/day) rate in 2011; in 2018, NL had the highest (10.1 DDD/1000/day) and BC had the lowest opioid dispensing rate (5.1 DDD/1000/day). Between 2011 and 2018, ON had the largest reduction (6.7 DDD/1000/day), whereas NL had a small increase (1.1 DDD/1000/day) in opioid dispensing; for ‘peak-to-2018’, ON had the largest reduction (6.7 DDD/1000/day) and QC had the smallest reduction (0.9 DDD/1000/day).

**Table 1:**
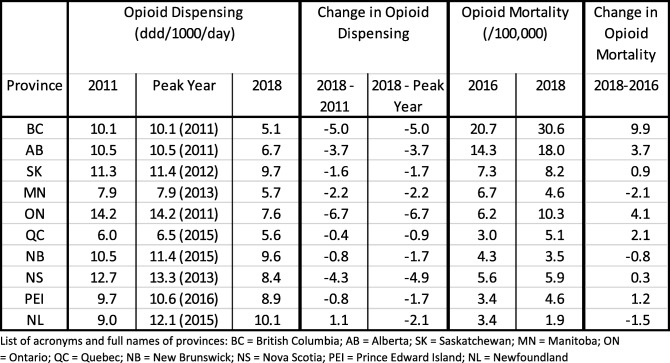
Annual opioid dispensing (1) 2018–2011 and (2) 2018 – ‘peak year’ and change rates, and opioid-related mortality (2018–2016) and change rates, by province in Canada.

List of acronyms and full names of provinces: *BC* British Columbia; *AB* Alberta; *SK* Saskatchewan; *MN* Manitoba; *ON* Ontario; *QC* Quebec; *NB* New Brunswick; *NS* Nova Scotia; *PEI* Prince Edward Island; *NL* Newfoundland

For opioid-related mortality, seven provinces had increases and three featured decreases in opioid-related mortality rates between 2016 and 2018. BC had the respective highest rates (20.7 deaths/100,000 population, 30.8/100,000, and 30.6/100,000) in all three years (2016–2018); the respectively lowest annual rates were QC (3.0/100,000), PEI (2.7/100,000) and NL (1.9/100,000). The largest increase in the opioid mortality rate was in BC (9.9/100,000), whereas the largest decrease was in MN (2.1/100,000) 2016–2018.

[See scatterplots in Fig. 1] The correlation between 1) changes in opioid dispensing (2011–2018) and changes in opioid-mortality (2016–2018) was 0.63 (df = 8, *p*-value = 0.05); for 2) the changes ‘peak year’-to-2018′ and corresponding changes in opioid-mortality (2016–2018) the correlation was 0.57 (df = 8, *p*-value = 0.09). Both sets of correlations were in the predicted direction; results were quasi-significant or borderline-significant, respectively.

## Discussion

In the above, we have shown quasi-significant correlations between recent (post-2010) changes in medical opioid dispensing and opioid-related mortality rates across Canada’s ten provinces. These findings come in a wider context of mostly substantial, yet provincially heterogeneous reductions in prescription opioid dispensing and equally extensive, yet equally heterogeneous changes – mostly by way of increases – in opioid-related mortality in Canada [1, 5]. The unprecedented increases in opioid overdose fatalities – outnumbering other major unnatural death categories and negatively impacting population-level life expectancy – have not been effectively addressed by interventions to date and require improved causal analyses towards improved intervention strategies [12, 22].

The recent major increases in opioid-related deaths have mainly been attributed to the sudden, pan-North American proliferation of potent and toxic illicit/synthetic opioid products (e.g., fentanyl and analogues). These illicit, mostly foreign produced opioid products, began to initially appear and boost opioid-related mortality in Canada (similar to the US, where increasing heroin use contributed to higher fatality rates) around 2015 [9, 23, 24]. These descriptive details, however, neither sufficiently explain the differential proliferation patterns, nor the highly differential contribution rates of these illicit opioid products to opioid-related mortality (range by province: 11–68% [2016]; 5–88% [2018]) across Canada [1].

Varying explanation approaches for the above developments exist. One is that illicit opioid supplies suddenly emerged on non-medical opioid markets as an independent supply ‘wave’ resulting in the described spikes of opioid mortality; this model however falls short of explaining the stark regional heterogeneity in illicit/synthetic opioid availability and their contributions to fatalities [13, 23]. A possibly different explanation – furthered elsewhere – is that illicit/synthetic opioid products proliferated as a wider substitution dynamic in response to reductions of and emerging gaps in medical opioid availability following various restrictive opioid control strategies implemented, with large populations of individuals previously exposed to (medical and non-medical) opioid use during previous periods of ample availability [23, 25]. At peak times (around 2010), > 20% of Canadians reported past-year use of prescription opioids, and > 5% were involved in non-medical use of opioids, translating into high levels of exposure in the general population [26]. Once the various opioid control measures became ramped up, large numbers of non-medical opioid users (including but not limited to use disorders) presumably needed to increasingly rely on both riskier (e.g., injecting) use practices as well as utilize new opioid supply sources – including illicit/synthetic opioid products - to meet their opioid demand needs, resulting in swiftly accelerating overdose and consequential fatality incidents [9, 15, 27].

Our results appear to at least initially corroborate and lend support to the latter explanation approach. Concretely, they support associations between the levels of province-based changes (mostly increases) in opioid-related mortality and changes (mostly reductions) in medical opioid availability post-2010. Or, differently put: opioid mortality tended to rise more strongly in jurisdictions that reduced medical opioid availability more substantially during the study period. While the results had only limited statistical significance, the associations’ consistent directionality and relative strength is notable given the small number of data points, specifically with only three years of opioid-mortality data available. Further noteworthy is that the observed decreases in opioid-mortality occurred exclusively in (three) provinces with later (i.e., post-2011) reductions in opioid availability, suggesting possible lag effects. Overall, our examinations should be extended towards more comprehensive and rigorous analyses, ideally with expanded data for the benefit of increased power and analytical strength.

Despite data and methods limitations, the results suggest several key implications: First, as shown elsewhere, supply dynamics for psychoactive drug use – especially where alternative (e.g., medical and non-medical sources) exist – appear to be both complex and dynamic; consequently, well-intended supply control measures can have un-intended, adverse consequences, including substitutions towards more hazardous substances [28]. Thus, in the context of the Canadian opioid crisis, recent, while differential reductions in medical opioid availability to reduce opioid-related health harms implemented post-2010 may have resulted in supply gaps subsequently filled by hazardous, illicit opioid products and consequential mortality increases. This furthermore implies that the existing, extensive ‘demand side’ for non-medical opioid use – despite substantially expanded prevention and treatment measures – has not been addressed commensurately with opioid supply reductions [25, 29, 30]. Overall, the totality of measures undertaken to reduce opioid-related health harms in Canada – where such efforts occur in a complex, multi-level system environment involving both, and at times conflicting, federal and provincial jurisdictions – since-2010 may have resulted in as much harm as benefit, including sudden, substantial spikes of mortality from illicit/synthetic opioids; hence, it is imperative to better understand the direct and indirect impacts of intervention measures towards at least improved policy development and design in the future [22, 28, 31].

Within these diverging developments facilitating Canada’s opioid crisis, the concrete need to prevent opioid mortality among existent at-risk opioid users is acute and urgent. In current contexts, this principally requires effective measures to supply high-risk opioid users with safer (e.g., medical grade) opioid products to reduce illicit, toxic drug exposure and consequential fatality outcomes [12, 32, 33]. Small, locally limited ‘safer opioid’ distribution programs (e.g., in Vancouver: [34]) have been initiated; however, these provisions ought to be scaled-up widely towards reaching substantial proportions of at-risk users and reducing fatal overdose incidents. Notably, such public health emergency-based ‘safer opioid’ provisions are at direct odds with the system-level reductions in medical opioid dispensing undertaken; reconciling these efforts towards overall public health benefit requires improved system-coordination of general prevention (i.e., reasonably limited opioid availability) to reduce undue opioid exposure in the general population with targeted prevention measures of safer opioid provision to reduce illicit/toxic opioid exposure among sub-populations of at-risk opioid users.

## Conclusions

We provide evidence for possible, ecological associations between the extent of reductions in longstanding, high levels of medical opioid availability and levels of opioid mortality on provincial population-levels in Canada. Such a correlation would most likely be explained with illicit, toxic opioid products filling emerging gaps in overall opioid supply in light of persistently high demands for (medical or non-medical opioid use). While more detailed and rigorous analyses are needed to further confirm these possible mechanisms, our data underscore that a fine and complex balance towards overall improved public health urgently needs to be found between reasonable reductions in opioid dispensing for general prevention vis-à-vis effective, targeted prevention measures for existing at-risk opioid user populations in the present contexts of an unrelenting national opioid mortality crisis in Canada.

## Data Availability

The datasets analysed for the present study included data extracted from a commercial database (IQVIA Canada’s Compuscript) on medical pharmaceutical (including opioid) prescriptions in Canada, and national data on opioid-related mortality (Health Canada). IQVIA’s Compuscript data reporting on opioid prescriptions are commercially available on specific request from IQVIA Canada. National opioid-related mortality data used in this study is publically available from Health Canada through its public surveillance and information database (https://health-infobase.canada.ca/substance-related-harms). Details related to the analyses of the data can be provided by the authors on reasonable request.

## Abbreviations

WHO: World Health Organization
ATC: Anatomical Therapeutic Chemical
DDD: Defined Daily Doses

## Canadian Provinces

AB: Alberta
BC: British Columbia
MB: Manitoba
NB: New Brunswick
NL: Newfoundland and Labrador
NS: Nova Scotia
ON: Ontario
PEI: Prince Edward Island
QC: Quebec
SK: Saskatchewan
